# Fehl- und Desinformation zu Nahrungsergänzungsmitteln in den sozialen Medien: Risiken begrenzen, Chancen nutzen, Strukturen gestalten

**DOI:** 10.1007/s00103-025-04138-x

**Published:** 2025-10-11

**Authors:** Emma C. Gauch, Martin Smollich

**Affiliations:** Institut für Ernährungsmedizin, Universitätsklinikum Schleswig-Holstein, Ratzeburger Allee 160, 23538 Lübeck, Deutschland

**Keywords:** Wissenschaftskommunikation, Influencer, Gesundheitlicher Verbraucherschutz, Fake News, Ernährungsinformationen, Science communication, Influencers, Consumer health protection, Fake news, Nutrition information

## Abstract

Soziale Medien (SoMe) sind für viele Menschen eine wichtige Quelle für Informationen über Nahrungsergänzungsmittel (NEM); gleichzeitig werden hier potenziell gesundheitsgefährdende Fehl- und Desinformationen verbreitet. Inhalte zu NEM sind in der Regel ökonomisch motiviert, ohne dass dies für die Nutzer*innen transparent erkennbar wäre. Hinsichtlich des gesundheitlichen Verbraucherschutzes ist diese Situation sehr problematisch. Andererseits bieten SoMe sehr gute Möglichkeiten für die evidenzbasierte Aufklärung breiter Bevölkerungsgruppen über NEM-bezogene Nutzen und Risiken.

Während die Auswirkungen von SoMe auf Gesundheitsverhalten, Gesundheit und Ernährung der Nutzer*innen bereits gut untersucht sind, liegen zu NEM bisher nur sehr wenige spezifische Daten vor. Deshalb werden im vorliegenden Beitrag die Datenlage zu NEM-bezogenen Informationen in den SoMe zusammengefasst sowie spezifische Risiken und Chancen aus der Perspektive des gesundheitlichen Verbraucherschutzes identifiziert. Auf dieser Grundlage werden konkrete Handlungsempfehlungen abgeleitet, um NEM-spezifische Strukturen im Kontext von SoMe so zu gestalten, dass dies sowohl den Verbraucher*innen als auch den seriösen Herstellern von NEM zugutekommt.

## Abstract (en)

Social media is an important and popular source of information related to dietary supplements (DSs); at the same time, social media also spreads potentially harmful misinformation and disinformation. Content on DSs is usually economically motivated without this being transparently recognizable to users. This situation is highly problematic in terms of consumer health protection. Nevertheless, social media offers excellent opportunities for evidence-based education of the population about DS-related benefits and risks.

While the effects of social media information on users’ health behavior, health, and nutrition have been well studied, there is a lack of specific data on DSs. This article therefore summarizes the data available on DS-related information on social media and identifies specific risks and opportunities from the perspective of consumer health protection. On this basis, concrete recommendations for action are derived that should serve to shape DS-specific structures in the context of social media that benefit both consumers and reputable DS manufacturers.

## Einleitung

Das Angebot an Nahrungsergänzungsmitteln (NEM) ist für viele Verbraucher*innen unüberschaubar. Und die Situation der NEM-bezogenen Informationen in den sozialen Medien (SoMe) ist aus Sicht des gesundheitlichen Verbraucherschutzes sehr problematisch: Die dort verbreiteten NEM-bezogenen Informationen sind in der Regel ökonomisch motiviert – oft durch Influencer*innen vermittelt – und von Fehl- und Desinformationen durchsetzt, während SoMe zugleich immer häufiger als Quelle für Gesundheitsinformationen genutzt werden [1–4].

Parallel dazu empfindet der Großteil der Menschen den Umgang mit gesundheitsrelevanten Informationen als zunehmend herausfordernd. So konnte in einer repräsentativen Studie gezeigt werden, dass 76 % der Internet-nutzenden Bevölkerung in Deutschland eine geringe Gesundheitskompetenz besitzen; 82 % verfügen nicht über ausreichende Fähigkeiten, um gesundheitsbezogene Informationen angemessen zu beurteilen [5]. Die Auswirkungen von SoMe-Informationen auf das Gesundheitsverhalten, die Gesundheit und die Ernährung der Nutzer*innen wurden in verschiedenen Studien untersucht [6–8]. Im Gegensatz dazu gibt es bisher nur sehr wenige spezifische Daten zu NEM.

Ziel des vorliegenden Beitrags ist es, die Datenlage zu NEM-bezogenen Informationen in SoMe zusammenzufassen, Risiken und Chancen aus der Perspektive des gesundheitlichen Verbraucherschutzes zu identifizieren und daraus konkrete Handlungsempfehlungen abzuleiten. Dazu wird zunächst auf die Relevanz von Fehl- und Desinformationen in SoMe sowie die Rolle von Influencer*innen eingegangen, bevor Ursprung, Ausprägungen und Folgen NEM-bezogener Fehlinformationen dargestellt und abschließend Handlungsempfehlungen formuliert werden.

## Fehl- und Desinformation in sozialen Medien – Relevanz und Mechanismen

Zu den aktuellen Herausforderungen des gesundheitlichen Verbraucherschutzes gehört die zunehmende Verbreitung von Fehl- und Desinformationen in SoMe. Die Begriffe „Fehlinformation“ und „Desinformation“ werden oft synonym verwendet, bezeichnen jedoch unterschiedliche Phänomene: Während „Fehlinformation“ falsche oder irreführende Inhalte beschreibt, die ohne Täuschungsabsicht verbreitet werden, steht „Desinformation“ für gezielt verbreitete Unwahrheiten mit der Absicht, einzelne Menschen oder Gruppen zu täuschen oder zu beeinflussen [9]. Der entscheidende Unterschied liegt damit in der Intention des Verbreitenden. In der Praxis ist es deshalb schwierig bis unmöglich, konkrete Einzelaussagen eindeutig als Fehl- oder Desinformation zu klassifizieren, da die Intention des Verbreitenden unbekannt ist und höchstens vermutet werden kann. Obwohl die Begriffe Fehl- und Desinformation aus dem sozial- bzw. politikwissenschaftlichen Kontext stammen, können sie auch auf die aktuelle Verbreitung von Gesundheits- und Ernährungsinformationen in SoMe angewendet werden.

### Verbreitung von Fehl- und Desinformation zu Gesundheit und Ernährung

Informationen zu NEM befinden sich an der Schnittstelle von Gesundheits- und Ernährungsinformationen. In beiden Bereichen haben SoMe die Mechanismen der Informationsverbreitung erheblich verändert [10]. Da es zentrales Strukturmerkmal der SoMe ist, dass Inhalte niedrigschwellig sowie unabhängig von Fachwissen und formaler Qualifikation veröffentlicht und verbreitet werden können, sind Nutzer*innen einer Informationsflut mit erheblichen Qualitätsunterschieden ausgesetzt [11].

Dementsprechend verläuft die Verbreitung von Fehl- und Desinformationen über SoMe rasant und hat weitreichende Auswirkungen auf unterschiedlichste gesellschaftliche Bereiche [12]. Dies betrifft auch die Themenbereiche Ernährung und Gesundheit, in denen Fehlinformationen häufig populärer sind als evidenzbasierte Inhalte [11]. Viele dieser Fehlinformationen stammen von Einzelpersonen, die weder fachlich qualifiziert sind noch im Namen einer Institution agieren. Die Narrative sind dabei oft durch eine persönliche, emotionale und negative Tonalität geprägt, die Angst, Unsicherheit und Misstrauen gegenüber wissenschaftlichen Institutionen und offiziellen Empfehlungen schüren können [11, 13]. Dadurch werden nicht nur gesundheitsbezogene Fehlinformationen verbreitet, sondern darüber hinaus wird das Vertrauen in seriöse Informationsquellen geschwächt. Ein prägnantes Beispiel für die praktischen Konsequenzen ist der gut dokumentierte Zusammenhang zwischen Fehlinformationen in SoMe und Impfskepsis im realen Leben [7].

### Fehl- und Desinformationen: Warum werden sie verbreitet und geglaubt?

Viele SoMe-Nutzer*innen besitzen weder das Fachwissen noch die digitale Gesundheitskompetenz, um gesundheitsbezogene Inhalte kritisch einordnen und reflektieren zu können [14]. Gleichzeitig aktivieren SoMe-typische Strukturen spezifische verhaltenspsychologische Mechanismen: Likes, Shares und andere Formen von sichtbaren Feedbacks verstärken den Eindruck sozialer Bewährtheit (*Social Proof*) von Informationen – insbesondere vor dem Hintergrund empfundener Unsicherheit und Angst. Inhalte, die oft geteilt oder positiv bewertet werden, wirken vertrauenswürdiger, selbst wenn sie objektiv falsch sind [15].

Ein Großteil der in SoMe geteilten falschen Informationen wird nicht gezielt als Desinformation verbreitet, sondern in der irrtümlichen Annahme, es handele sich um Fakten. Kontroverse Aussagen erzielen dabei eine besonders hohe Reichweite, woraus sich eine selbstverstärkende Dynamik ergibt [14]. Umgekehrt konnte gezeigt werden, dass sich die Verbreitung von Fehlinformationen um 51 % reduziert, wenn die Nutzer*innen vorab aufgefordert werden, innezuhalten und die Richtigkeit der Inhalte zu reflektieren [16]. Kritisches Denken hilft jedoch nicht automatisch, Informationen richtig einzuordnen, sondern kann auch in pauschales Misstrauen gegenüber sämtlichen Informationsquellen umschlagen [17].

Entscheidende Prädiktoren für die Einschätzung des Wahrheitsgehalts gesundheitsbezogener Aussagen in SoMe sind die Art der Quelle, die inhaltliche Glaubwürdigkeit und das Design (*Look and Feel*) der Beiträge [18].

Ein weiteres systemimmanentes Problem von SoMe ist die Künstliche-Intelligenz-(KI-) bzw. algorithmusbasierte Inhaltsverbreitung in Kombination mit Internet-Robots (Bots) und „Filter-Bubble“-Mechanismen: Beiträge mit hohem Interaktionspotenzial erhalten dadurch mehr Sichtbarkeit – unabhängig von ihrem Wahrheitsgehalt [13]. Da provokante oder emotional aufgeladene Inhalte überdurchschnittlich viele Interaktionen erzeugen, begünstigen die zugrunde liegenden Algorithmen beispielsweise auf TikTok die Verbreitung entsprechender Fehl- und Desinformationen [19]. Demnach gehört zur Informationskompetenz in Zeiten von SoMe nicht mehr nur die klassische Quellenkritik, sondern auch ein Bewusstsein für und ein strategischer Umgang mit Algorithmen [20].

### Influencer*innen und parasoziale Beziehungen

Hinzu kommt ein weiterer Aspekt: Durch das strukturelle Design der SoMe sind die Hürden für die Content-Erstellung minimal, sodass Nutzer*innen motiviert werden, eigene Inhalte zu generieren. Personen, denen es durch ihre SoMe-Beiträge gelingt, eine große Reichweite zu erzielen und damit das Kaufverhalten und die Meinungen ihrer Follower*innen zu beeinflussen, werden als Influencer*innen bezeichnet [21]. Charakteristisch für das Phänomen der Influencer*innen ist das hohe Maß an Glaubwürdigkeit und Vertrauen, das ihre Follower*innen ihnen entgegenbringen [22]. Wesentlicher Prädiktor für die empfundene Glaubwürdigkeit gesundheitsbezogener SoMe-Inhalte ist neben dem formalen Expert*innen-Status des Absenders und dem Sprachstil (persönlich vs. neutral) der Mitläufereffekt („Bandwagon-Heuristik“); demnach steigt die empfundene Glaubwürdigkeit mit der Zahl der Follower*innen, der Likes und der Shares [23]. Aufgrund dieser kommunikationspsychologischen Konstellation ist es möglich, dass auch Influencer*innen ohne jede formale oder fachlich begründete Expertise die Werturteile und Kaufentscheidungen ihrer Follower*innen maßgeblich beeinflussen [24].

Integraler Bestandteil dieses weitreichenden Einflusses von Influencer*innen sind die parasozialen Beziehungen, die sie zu ihren Follower*innen aufbauen, indem sie durch Fotos, Videos und Storys kontinuierliche Einblicke in ihr Leben gewähren. Als „Story“ wird bei Instagram eine Funktion beschrieben, mit der Fotos oder Videos geteilt werden können, die dann nur für 24 h sichtbar bleiben. Unter parasozialen Beziehungen versteht man in diesem Kontext einseitige emotionale und soziale Verbindungen, die Mediennutzer*innen zu medialen Persönlichkeiten aufbauen und die als ebenso bedeutsam empfunden werden können wie reale Partnerschaften [25, 26]. Die Möglichkeit, durch Likes und Kommentare mit Influencer*innen zu interagieren, verstärkt das Gefühl der Nähe und lässt die Beziehung wechselseitig erscheinen [26, 27].

Das Vertrauen, die Glaubwürdigkeit und die als freundschaftsähnlich empfundene parasoziale Beziehung machen Influencer*innen zu wichtigen Meinungsführer*innen. Die von ihnen geteilten Produktempfehlungen und Informationen werden von den jeweiligen Follower*innen als sehr glaubwürdig eingestuft und kaum kritisch hinterfragt [28, 29]. Das betrifft auch Empfehlungen und Bewertungen von NEM.

## NEM-bezogene Fehl- und Desinformationen in sozialen Medien

Obwohl die Auswirkungen von SoMe-Informationen auf das Gesundheits- und Konsumverhalten von Verbraucher*innen inzwischen sehr gut untersucht sind, fehlen spezifische Daten zu NEM-bezogenen SoMe-Inhalten weitestgehend. Dennoch kann davon ausgegangen werden, dass aus den wenigen publizierten Studienergebnissen und den allgemeinen Effekten von SoMe auf die Gesundheit und die Ernährung der Nutzer*innen gewisse Schlussfolgerungen gezogen werden können.

### Informationsquellen und Kommunikationspraktiken zu NEM in sozialen Medien

Bezüglich der Informationslandschaft zu NEM in SoMe sind zunächst 3 Aspekte von besonderem Interesse: die Relevanz von SoMe als Informationsquelle, die Akteur*innen, die entsprechende Inhalte verbreiten, sowie die jeweiligen Kommunikationsformen.

Systematische Untersuchungen zur Relevanz von SoMe für NEM-bezogene Informationen fehlen bislang; allerdings liefern einzelne Studien gewisse Anhaltspunkte. So konnte in einer US-amerikanischen Untersuchung gezeigt werden, dass sich über 80 % der Befragten vor der Einnahme von NEM im Internet informierten; 41 % der Befragten nutzten das Internet sogar als ausschließliche Informationsquelle [30]. Herkömmliche Ansprechpersonen wie Apotheker*innen, Ernährungsfachkräfte oder Ärzt*innen wurden hingegen deutlich seltener konsultiert. Ähnliche Ergebnisse lieferte eine Studie aus dem Libanon: Unter 455 befragten Athlet*innen wurden Trainer*innen (74 %) am häufigsten als Informationsquelle zu NEM genannt, gefolgt von Online-Plattformen, inklusive SoMe (64 %) [31]. Gesundheitsfachkräfte spielten hier ebenfalls nur eine nachrangige Rolle. Offen ist allerdings, inwieweit diese Studienergebnisse auf die Allgemeinbevölkerung übertragbar sind. In Bezug auf Informationen zu Ernährungsthemen wurde in einer Studie gezeigt, dass 72 % der 18- bis 49-Jährigen in Deutschland SoMe als eine Informationsquelle nutzen; allerdings wurde hierbei nicht spezifisch nach NEM gefragt [32]. Dabei wurde YouTube als wichtigste Plattform für Ernährungsinformationen genannt (40 %), gefolgt von Facebook (21 %) und Instagram (20 %); Pinterest (9 %) und TikTok (4 %) lagen deutlich dahinter.

Ein wichtiger Aspekt in der Gesundheits- und Ernährungskommunikation in SoMe ist die Absenderqualifikation. Forschung zu diesem Thema zeigt, dass die Inhalte häufig nicht von qualifizierten Fachpersonen stammen [19, 33]. Gleichzeitig bewerten Nutzer*innen SoMe-Informationen maßgeblich danach, wie ansprechend und nahbar die Inhalte aufbereitet sind [32, 34]. Dies führt zu einer Verzerrung der Informationslandschaft, in der emotionale und visuell attraktive Inhalte bevorzugt werden – unabhängig von ihrer inhaltlichen Qualität [3, 19, 33]. Im Vergleich zu fachlich nicht qualifizierten Influencer*innen sind Expert*innen mit formaler Qualifikation sowie offizielle Organisationen aus dem Bereich Ernährung und Gesundheit in den SoMe deutlich weniger präsent. Zwar ist die Mehrheit der öffentlichen Gesundheitsinstitutionen inzwischen aktiv auf verschiedenen SoMe-Plattformen vertreten [38]; dabei entsprechen die am häufigsten genutzten Plattformen jedoch nicht unbedingt den bevorzugten Plattformen der Zielgruppen: Insbesondere TikTok ist hierbei stark unterrepräsentiert, obwohl dies die Plattform mit der größten Relevanz für junge Menschen ist [35].

Daneben spielt die Art der Kommunikation eine entscheidende Rolle. Gesundheitsorganisationen kommunizieren in SoMe überwiegend sachlich und unpersönlich [34, 36, 37], obwohl ein plattformgerechter Kommunikationsstil durch Emotionalität, Subjektivität und Interaktivität charakterisiert ist [23]. Ein weiterer zentraler Unterschied liegt im Ziel und Geschäftsmodell der jeweiligen Akteur*innen: Während Gesundheitsorganisationen ihre SoMe-Kommunikation auf Public-Health-Aspekte ausrichten, verfolgen Influencer*innen ganz überwiegend kommerzielle Interessen [3, 34, 37]. Die Informationsvermittlung wird dabei als Teil einer Marketingstrategie für Produkte genutzt, indem nicht der Hersteller selbst Werbeaussagen tätigt, sondern Influencer*innen im Rahmen von Kooperationen, die häufig nicht transparent gemacht werden. Das erschwert die Unterscheidung zwischen der von der Meinungsfreiheit gedeckten Fehlinformation und der ökonomisch motivierten Desinformation bzw. macht sie sogar unmöglich [2, 38]. Das Ausmaß des kommerziellen Contents wird beispielsweise in einer Analyse von 1000 Instagram-Beiträgen deutschsprachiger Influencer*innen zu den Themen „dieting“ und „exercise“ verdeutlicht: In 71 % der Posts war mindestens eine Marke präsent [3]; NEM stellten dabei mit 75 % den am häufigsten beworbenen Produktbereich dar. Auch in plattformübergreifenden Untersuchungen konnte gezeigt werden, dass NEM-bezogene Inhalte im Vergleich zu anderen gesundheitsbezogenen Themenfeldern besonders häufig mit werblicher Kommunikation verknüpft sind [4]. Eine aktuelle Untersuchung unter Jugendlichen ergab, dass mehr als die Hälfte der Befragten in den letzten 6 Monaten ein Produkt gekauft hat, das von einem Influencer oder einer Influencerin beworben wurde.^1^

Inwieweit sich die verschiedenen SoMe-Plattformen hinsichtlich der auf ihnen geteilten NEM-bezogenen Informationen unterscheiden, ist nicht systematisch untersucht. In einem aktuellen Review wurden Instagram (50 %) und YouTube (39 %) als die am häufigsten genutzten Plattformen zur Verbreitung von ernährungsbezogenen Fehlinformationen identifiziert [39]. Ob dies in vergleichbarer Weise für NEM-bezogene Fehl- und Desinformationen gilt, ist unklar.

### Irreführende und intransparente Inhalte

Eine häufige Form NEM-bezogener Fehl- bzw. Desinformation besteht darin, dass wesentliche Angaben zu Inhaltsstoffen, Mengenangaben sowie erforderliche Warnhinweise fehlen [2, 40]. In einer Analyse der meistgesehenen YouTube-Videos zu Multinährstoffpräparaten wurden in 84 % der Beiträge die potenziellen Risiken im Zusammenhang mit der Einnahme des jeweiligen Produkts nicht erwähnt [41].

Zusätzlich problematisch ist die häufig unzureichende Kennzeichnung von Werbung durch Influencer*innen. In einer Analyse deutschsprachiger SoMe-Accounts waren nur 6 % der Beiträge als Werbung gekennzeichnet, obwohl in 62 % der Beiträge ein Produkt oder eine Marke explizit genannt wurden [42]. Eine Analyse von TikTok-Videos zu Gewichtsverlust‑, Muskelaufbau- und sog. Detox-Präparaten zeigte, dass in 95 % der Fälle nicht offengelegt wurde, ob es sich um bezahlte Produktplatzierungen handelte [1]. Dass eine transparente Werbekennzeichnung die Wirkung werblicher Inhalte deutlich abschwächen kann, konnte vielfach gezeigt werden [43]. Zudem erhielten Beiträge mit Markenpräsenz, die nicht als Werbung gekennzeichnet waren, mehr Likes und Kommentare als entsprechend gekennzeichnete Beiträge [1, 44]. Insgesamt verstärkt fehlende Werbekennzeichnung also die Wirkung und Glaubwürdigkeit ökonomisch motivierter NEM-Beiträge. Das ist insofern relevant, als bezahlte Beiträge oft deutlich häufiger positive Effekte von NEM betonen als unbezahlte [38]. Außerdem enthalten Beiträge mit finanziellen Interessen im medizinischen Kontext seltener Hinweise auf mögliche Risiken der beworbenen Produkte [33].

Häufig werden NEM in SoMe wissenschaftlich nicht belegte gesundheitsbezogene Effekte zugeschrieben [1, 40]; dies betrifft unter anderem NEM mit vermeintlich „entgiftenden“, gewichtsreduzierenden und sog. „Beauty“-Wirkungen [1, 2]. In einer Analyse von 187 Pinterest-Beiträgen mit Aussagen zur Prävention und Therapie von Brustkrebs enthielten 51 % Fehlinformationen, wovon sich 19 % auf NEM bezogen [45]. In einer Untersuchung des Chemischen und Veterinäruntersuchungsamts Stuttgart (CVUA) wurden 90 % der Werbeaussagen von Influencer*innen auf Instagram als unzulässig gemäß der europäischen Health-Claims-Verordnung (HCVO) eingestuft.^2^

### Risiken von Fehl- und Desinformationen

Der Zusammenhang zwischen individuellem Wissen und der Anfälligkeit für Fehl- und Desinformation im Gesundheitskontext ist gut belegt. Im Zuge einer Untersuchung des Bundesinstituts für Risikobewertung (BfR) konnte gezeigt werden, dass sich 35 % der Befragten „gar nicht“/„nicht gut“ über das gesundheitliche Risiko von vitaminhaltigen NEM informiert fühlen [46]. Dabei fällt es Personen mit geringem wissenschaftlichen Grundwissen deutlich schwerer, wahre von falschen Informationen zu unterscheiden [14, 47]. Dies führt zu einer problematischen Situation: Obwohl das Fachwissen über NEM in der Allgemeinbevölkerung sehr gering ist, überschätzen viele ihre eigene Kompetenz erheblich [31, 48]. Diese Diskrepanz zeigt sich ebenfalls im Zusammenhang zwischen Wissensstand und Werbewirkung: Personen ohne medizinische Vorbildung glauben NEM-bezogene Werbeaussagen signifikant häufiger als medizinisch geschulte Befragte [48]. Es ist daher davon auszugehen, dass ein erheblicher Teil der Bevölkerung anfällig für NEM-bezogene Fehl- und Desinformationen in SoMe ist.

Auf diese Weise können SoMe-Inhalte dazu beitragen, ärztliche Empfehlungen zu relativieren: In einer Studie zu Akne-Patientinnen beispielsweise suchte ein Großteil der Befragten in SoMe nach Informationen und folgte den dort gegebenen Empfehlungen – einschließlich der Einnahme von NEM [49]. Dabei veränderten 20,9 % eigenständig ihre ärztlich verordnete Behandlung auf Grundlage dieser SoMe-Inhalte. Ein weiteres Gesundheitsrisiko besteht darin, dass NEM, wie beispielsweise Kollagen, mit unbelegten Heilsversprechen beworben werden – etwa in Bezug auf Schlaf, Verdauung oder Herz-Kreislauf-Gesundheit – und dies zur Vernachlässigung bzw. zum Abbruch wirksamer medizinischer Therapien zugunsten unwirksamer NEM führen könnte [38].

Neben den gesundheitlichen Risiken NEM-bezogener Fehl- und Desinformationen ergeben sich häufig auch ökonomische Nachteile für Konsument*innen, da Informationen zu NEM auf SoMe häufig in einen werblichen Kontext eingebettet sind [3, 4]. Preisangaben zu den beworbenen NEM fehlen in aller Regel und der weitverbreitete Einsatz von Rabattcodes animiert gezielt zum Kauf [40]. Eine diesbezüglich besonders vulnerable Zielgruppe des NEM-Marketings sind Menschen mit Krebserkrankungen, da die Prävalenz der NEM-Einnahme in dieser Personengruppe höher ist als in der Gesamtbevölkerung: Beispielsweise wurde gezeigt, dass fast die Hälfte aller onkologischen Patient*innen NEM einnehmen [50, 51]. Die onkologische Diagnose führt an dieser Stelle zu einem deutlichen Anstieg der NEM-Einnahme: In einer aktuellen Befragung von 320 Brustkrebs-Patient*innen gaben 20,2 % der Befragten an, vor der Diagnose NEM eingenommen zu haben; nach der Diagnose waren es 64,4 % der Befragten [52].

## Chancen der NEM-bezogenen Social-Media-Kommunikation

Trotz der genannten Risiken müssen SoMe im Kontext gesundheits- und NEM-bezogener Informationen differenziert bewertet werden; beispielsweise können SoMe durchaus als sehr effektive Kanäle zur Verbreitung evidenzbasierter NEM-Informationen und als soziale Unterstützung bei der Verfolgung individueller Gesundheitsziele dienen [53, 54]. Durch die SoMe-typischen Kommunikationsformen können medizinische Themen komplexitätsreduziert und zielgruppengerecht aufbereitet und dadurch einfacher zugänglich gemacht werden [32]. Gleichzeitig können parasoziale Beziehungen als erhebliche Motivationssteigerung für gesundheitsförderliches Verhalten genutzt werden und das subjektive Gefühl von Selbstbestimmung im Umgang mit gesundheitlichen Herausforderungen stärken. So können Influencer*innen ihre Reichweite nutzen, um zur Aufklärung über NEM-bezogene Chancen und Risiken beizutragen. In einer Analyse von TikTok-Beiträgen mit dem Hashtag *#dietpills* konnte beispielsweise gezeigt werden, dass rund die Hälfte der Videos explizit kritisch im Ton war, vor den Risiken der Präparate warnte und fachlich korrekte Informationen lieferte [1]. Ein Teil dieser Beiträge betonte außerdem die Relevanz gesunder Ernährung und Bewegung.

Gerade Personen mit chronischen Erkrankungen besitzen ein ausgeprägtes NEM- und ernährungsbezogenes Informationsbedürfnis, für das der überwiegende Teil der Betroffenen SoMe als Informationsquelle nutzt [55, 56]. Dementsprechend können SoMe ein geeigneter Kanal sein, um diesen Informationsbedarf zielgruppengerecht zu adressieren und für die evidenzbasierte Wissenschaftskommunikation zu nutzen. Dadurch ließen sich möglicherweise gesundheitliche Risiken durch NEM-Einnahme reduzieren, da viele Patient*innen bezüglich der Einnahme von NEM keinen ärztlichen Rat einholen, sondern sich stattdessen eigenständig, unter anderem über SoMe, informieren [57].

## Ableitung von Vorschlägen zur Verbesserung der Situation

Die NEM-bezogenen Informationen auf SoMe sind sowohl mit Risiken als auch mit Chancen verbunden. Vor diesem Hintergrund sollen die folgenden Handlungsempfehlungen dazu dienen, die Verbreitung von NEM-bezogenen Fehl- und Desinformationen einzudämmen und gleichzeitig die gesundheitsförderlichen Potenziale der SoMe zu nutzen.

### Verstärkte, proaktive SoMe-Nutzung durch öffentliche Gesundheitseinrichtungen und Fachgesellschaften.

Die Art und Aufmachung der Inhalte sollte sich dabei nicht an der etablierten Kommunikationskultur des Senders, sondern am Mediennutzungsverhalten des Empfängers (der Zielgruppe) orientieren [58]. Um die Reichweite zu sichern und die Bindung der Follower*innen zu festigen, sind häufige Aktualisierungen der Informationsangebote und möglichst viele interaktive Elemente essenziell. Statische Websites sind hierfür wenig geeignet [59]. Dazu ist ein grundlegender Mentalitätswandel erforderlich, da die traditionelle Kommunikationskultur insbesondere von Behörden überwiegend einseitig gerichteten Sender-Empfänger-Strukturen folgt und kaum auf Agilität und soziale Interaktionen ausgelegt ist [60]. Sinnvoll ist außerdem die Kooperation von Fachgesellschaften und offiziellen Stellen mit professionellen Influencer*innen, um deren Reichweite und Glaubwürdigkeit zu nutzen; dies ist zumindest am Anfang aussichtsreicher als der Aufbau eigener SoMe-Accounts durch Behörden [36, 61].

### Orientierung der NEM-bezogenen Gesundheitskommunikation an den etablierten SoMe-Strategien der Lifestyle- und Supplementindustrie.

Die Inhalte sollten zwar fachlich fundiert, aber trotzdem persönlich, emotional und mit positiver Tonalität präsentiert werden. Dazu gehört neben der zielgruppen- und plattformgerechten Aufbereitung der Inhalte eine klare und leicht verständliche Sprache [34]. Da die Glaubwürdigkeit von SoMe-Inhalten überwiegend nach intuitiv schnell erfassbaren Kriterien bewertet wird, muss visuellen Elementen besondere Beachtung geschenkt werden. Textbasierte Informationsvermittlung ist hierfür weniger geeignet. Zum Aufbau wirksamer parasozialer Beziehungen sollten die Informationen nicht durch abstrakte Institutionen vermittelt werden, sondern durch emotional zugängliche Personen.

### Zeitnahes und systematisches Widerlegen (Debunking) von NEM-bezogenen Fehl- und Desinformationen.

Korrekturen von Fehl- und Desinformationen durch Fachexpert*innen sind deutlich wirksamer als durch Laien [62]. Aufgrund der kommunikativen SoMe-Dynamik ist eine zeitnahe Reaktion entscheidend, sodass behördeninterne Abspracheverfahren und Abläufe angepasst werden müssen. Grundlage dieses Debunking sollte idealerweise ein systematisches Screening der verschiedenen Plattformen auf Fehl- und Desinformationen sein. Aus Effizienzgründen wäre es dabei zielführend, wenn sowohl für das Screening als auch für die kurzfristige Reaktion auf NEM-bezogene Fehl- und Desinformationen die Expertise von Aufsichtsbehörden und Fachgesellschaften in einer zentralen Einrichtung (*Fact Checking*) gebündelt wird. Diese Einrichtung sollte fachlich und finanziell unabhängig sein und könnte als universitäres Institut angelegt werden.

### Motivation und Qualifizierung von Ärzt*innen, Ernährungsfachkräften und Angehörigen anderer Gesundheitsfachberufe für die fachbezogene SoMe-Nutzung.

Angehörige von Heilberufen und Wissenschaftler*innen besitzen neben ihrer fachlichen Expertise und Praxiserfahrung einen hohen Vertrauensvorschuss in der Bevölkerung. Diese Menschen sollten deshalb systematisch dabei unterstützt werden, sich in den SoMe mit evidenzbasierter Aufklärung zu positionieren [19]. Hierbei können entsprechende Fort- und Weiterbildungsangebote der Fachgesellschaften und Berufsverbände sowie die Unterstützung durch Pressestellen des jeweiligen Arbeitgebers hilfreich sein.

### Einführung eines Qualitätssiegels für Influencer*innen, deren NEM- bzw. gesundheitsbezogenen Inhalte fachlich fundiert sind.

Influencer*innen und SoMe-Accounts, die fachlich korrekte Informationen zu NEM bzw. zu medizinischen Themen insgesamt präsentieren (sog. Medfluencer), könnten durch ein spezifisch definiertes Qualitätssiegel ausgezeichnet werden. Damit würde die besondere Glaubwürdigkeit der Inhalte für Laien erkennbar. Die Anforderungen eines solchen Qualitätssiegels könnten durch Fachgesellschaften definiert und die Akkreditierung an ein unabhängiges Institut übertragen werden.

### Personelle und strukturelle Stärkung von Lebensmittelüberwachung und Verbraucherschutz.

Die NEM-bezogenen Verstöße gegen die Health-Claims-Verordnung und andere gesetzliche Vorgaben auf SoMe-Plattformen werden oft deshalb nicht verfolgt und geahndet, weil die personellen und strukturellen Ressourcen der entsprechenden Behörden unzureichend sind. Eine Verbesserung dieser Situation wäre eine wichtige verbraucherschutzpolitische Aufgabe.

Diese Handlungsempfehlungen sollten idealerweise durch gesundheits- und bildungspolitische Maßnahmen flankiert werden [63]. Dazu gehören einerseits die grundsätzliche Verbesserung der Medien- und Gesundheitskompetenz der Bevölkerung und die Förderung von Verbraucherbildung. Andererseits gäbe es Maßnahmen, deren Umsetzung bei den Betreibern der SoMe-Plattformen liegt. Dies sind insbesondere Anpassungen des Algorithmus, um evidenzbasierte Inhalte stärker auszuspielen, Fact-Checking-Initiativen und einordnende Kommentare sowie automatisierte Aufforderungen zur Reflexion, bevor zweifelhafte Inhalte geteilt werden [14, 19, 60, 64]. Gerade der letztgenannte Punkt würde auch die Eigenverantwortung der SoMe-Nutzer*innen hinsichtlich der Verbreitung von Fehl- und Desinformationen betonen und unterstützen. Bei der Einwirkung auf die Betreiber der SoMe-Plattformen müssen politische Gestaltungsmöglichkeiten genutzt werden. Auch die Förderung von Angeboten wie „Klartext Nahrungsergänzung“ der Verbraucherzentrale Nordrhein-Westfalen gehört zu den Möglichkeiten, wie evidenzbasierte NEM-bezogene Informationen niedrigschwellig und allgemeinverständlich zugänglich gemacht und für die Verbraucherbildung genutzt werden können.

Abschließend soll ein wichtiger Punkt nicht unerwähnt bleiben: Von der politisch unterstützen Realisierung der hier vorgestellten Handlungsempfehlungen würden nicht nur die Verbraucher*innen profitieren; vielmehr würden damit auch all jene NEM-Hersteller unterstützt, die seriösen Geschäftspraktiken folgen und die mit hochwertigen und sicheren Produkten am Markt vertreten sind.

## Fazit

Soziale Medien (SoMe) sind für viele Menschen eine wichtige Quelle für Informationen zu Nahrungsergänzungsmitteln (NEM); gleichzeitig werden hier potenziell gesundheitsgefährdende Fehl- und Desinformationen verbreitet. Mit einer freiwilligen Regulierung durch die Plattformbetreiber ist in absehbarer Zeit nicht zu rechnen. Andererseits bieten SoMe sehr gute Möglichkeiten für die evidenzbasierte Aufklärung breiter Bevölkerungsgruppen über NEM-bezogene Nutzen und Risiken. Um diese Ziele zu erreichen und seriöse NEM-Hersteller zu unterstützen, sollten die abgeleiteten Handlungsempfehlungen mit politischer Unterstützung umgesetzt werden. Gleichzeitig besteht weiterer Forschungsbedarf hinsichtlich der gesundheitlichen Effekte NEM-bezogener Fehl- und Desinformationen in SoMe, insbesondere differenziert nach verschiedenen Bevölkerungsgruppen und unterschiedlichen SoMe-Plattformen.

## Footnotes

1 https://www.isi.fraunhofer.de/de/presse/2024/presseinfo-03-social-media-influencer-marketing-jugendliche-kritischer-konsum-schutz-noetig.html, letzter Zugriff 25.06.2025.

2 https://www.ua-bw.de/pubmobil/beitrag.asp?Thema_ID=2&ID=3577&subid=1, letzter Zugriff 25.06.2025.
